# Development of a scale to measure stigma related to podoconiosis in Southern Ethiopia

**DOI:** 10.1186/1471-2458-13-298

**Published:** 2013-04-04

**Authors:** Hannah Franklin, Abebayehu Tora, Kebede Deribe, Ayalu A Reda, Gail Davey

**Affiliations:** 1 Brighton and Sussex Medical School, Brighton, UK; 2 Department of Sociology, Wolita Sodo University, Sodo, Ethiopia; 3 Department of Public Health, College of Health Sciences, Haramaya University, Harar, Ethiopia

**Keywords:** Stigma, Measure, Podoconiosis, Neglected tropical disease, Ethiopia, Validity

## Abstract

**Background:**

Health-related stigma adds to the physical and economic burdens experienced by people suffering from neglected tropical diseases (NTDs). Previous research into the NTD podoconiosis showed significant stigma towards those with the disease, yet no formal instrument exists by which to assess stigma or interventions to reduce stigma. We aimed to develop, pilot and validate scales to measure the extent of stigma towards podoconiosis among patients and in podoconiosis-endemic communities.

**Methods:**

Indicators of stigma were drawn from existing qualitative podoconiosis research and a literature review on measuring leprosy stigma. These were then formulated into items for questioning and evaluated through a Delphi process in which irrelevant items were discounted. The final items formed four scales measuring two distinct forms of stigma (felt stigma and enacted stigma) for those with podoconiosis and those without the disease. The scales were formatted as two questionnaires, one for podoconiosis patients and one for unaffected community members. 150 podoconiosis patients and 500 unaffected community members from Wolaita zone, Southern Ethiopia were selected through multistage random sampling to complete the questionnaires which were interview-administered. The scales were evaluated through reliability assessment, content and construct validity analysis of the items, factor analysis and internal consistency analysis.

**Results:**

All scales had Cronbach’s alpha over 0.7, indicating good consistency. The content and construct validity of the scales were satisfactory with modest correlation between items. There was significant correlation between the felt and enacted stigma scales among patients (Spearman’s r = 0.892; p < 0.001) and within the community (Spearman’s r = 0.794; p < 0.001).

**Conclusion:**

We report the development and testing of the first standardised measures of podoconiosis stigma. Although further research is needed to validate the scales in other contexts, we anticipate they will be useful in situational analysis and in designing, monitoring and evaluating interventions. The scales will enable an evidence-based approach to mitigating stigma which will enable implementation of more effective disease control and help break the cycle of poverty and NTDs.

## Background

Stigma is increasingly being recognised as an obstacle to good health and a barrier to accessing healthcare. Health-related stigma affects the life chances of individuals, increasing their exposure to risks and limiting access to protective factors, potentially adding to their burden of disease or disability [1]. Assessing levels of stigma is therefore becoming increasingly important for public health, particularly with regard to guiding policy, designing and evaluating interventions, and advocacy work. Research is required to both clarify the nature of the stigma burden and develop and test strategies for mitigating problematic stigma [2].

Health-related stigma is typically characterised by social disqualification of individuals and populations who are identified with particular health problems and judged as a result of that condition [2]. This stigma can be divided into enacted stigma and felt stigma [3]. Enacted stigma refers to the actual experience of discrimination, including both discriminatory acts, such as abuse or loss of employment, and prejudicial attitudes [4]. In contrast, felt (or perceived) stigma refers to fear of this enacted stigma [5]. This might involve individuals with a stigmatised condition purposely avoiding social situations to reduce opportunities for enacted stigma [3]. Further still, social judgement may not only be anticipated but internalised, so those who are stigmatised devalue themselves, and in turn feel a sense of shame [6]. Both felt and enacted stigma can pose threats to the self-esteem and security of those with certain diseases or symptoms [5] as well as further impacting on their health status.

The stigma surrounding neglected tropical diseases (NTDs) is particularly detrimental, fuelling insecurities which keep people trapped in a vicious cycle of poverty [6,7]. Podoconiosis is an example of an NTD caused by long-term exposure of bare feet to irritant volcanic soils with symptoms which include swelling of the lower leg and skin thickening. The stigma attached to the disease limits social and economic opportunities for affected individuals and thwarts efforts to effectively treat and prevent podoconiosis, negatively impacting on health-seeking behaviour and acting as a barrier to appropriate care from health workers [8-10].

In order to implement effective NTD control programmes, gauging the nature of stigma is required, alongside assessments of the physical environment and medical resources available [7]. Instruments to measure stigma can be used to assess the magnitude of NTD-related stigma in a community and identify which manifestations of stigma are most profound. Understanding the dynamics of podoconiosis stigma will enable the design of more effective interventions: the data collected may in future be used to evaluate and tailor stigma reduction strategies specific to community needs.

Although stigma related to podoconiosis has been explored quantitatively [8,9] and qualitatively [10,11], there is no validated tool to measure stigma. In addition, previous research did not differentiate between felt stigma and enacted stigma. The purpose of this study was therefore to develop, pilot and validate a scale to measure the extent of stigma towards podoconiosis, drawing from other NTD stigma scales and existing research on podoconiosis.

## Methods

### Development of the study tool

We planned to include items measuring felt stigma and enacted stigma at both the family and community level to encompass stigma found in day-to-day interactions and in wider social participation. We also aimed to include both those affected by podoconiosis and unaffected community members.

Potential items to be included in the scales were identified by analysing the findings of studies investigating community knowledge, attitudes and practices towards individuals with podoconiosis [8-11]. Other potential items were identified from a literature review on measuring stigma towards leprosy [12], another highly stigmatised NTD with symptoms that are hard to conceal, like podoconiosis. Since individuals with podoconiosis face similar exclusionary social treatment to those with leprosy, commonly used items in leprosy stigma assessments were also included in this study.

Potential items were then grouped according to the WHO International Classification of Functioning, Disability and Health (ICF) [13]. Of the nine domains, leprosy stigma had greatest impact on mobility, domestic life, interpersonal interactions and relationships, major life areas, and community, social and civic life [12]. Again, existing qualitative research [10,11] has demonstrated the effect podoconiosis stigma has on these areas, so these formed the basis through which stigma was assessed (Table 1).

**Table 1:** Indicators of podoconiosis stigma in three domains

**Domain 1: Interpersonal interactions (domestic life, family/neighbourhood relationships)**	**Domain 2: Major life areas**	**Domain 3: Community, social and civic life**
• Buying items at market	• Employment	• Leadership and decision making
• Eating/living separately	• Marriage	• Participation in community affairs, public events and social organisations
• Interactions with family, friends, neighbours and health professionals		
• Isolation from others		
• Shame/embarrassment		

Two structured 50-item pilot scales were produced based on the indicators in Table 1, one for podoconiosis patients and one for unaffected community members. The scales generated were first evaluated through a Delphi process in which six people with expertise in the area of podoconiosis were asked to rate the relevance of items included in the preliminary scales. After several discussions, an exclusion consensus was reached and items considered irrelevant were rejected. The inclusion criteria decided by the research team were that each item had to be universal (applicable in different cultural contexts and across different groups such as age, marital status and social class); had to measure stigma (rather than confidence, or self-esteem) and could be clearly categorised as felt or enacted stigma. On the basis of these criteria, 18 items were excluded from the preliminary podoconiosis patient scales.

The scales agreed for testing were formatted as questionnaires containing both positively and negatively framed items to address response set bias, and translated into both the Ethiopian national language (Amharic) and the local language (Wolaitigna) and back translated into English to check for consistency.

Drawing from responses used in scales to measure stigma towards onchocerciasis [14,15] participants were asked to respond with either ‘yes’, ‘possibly’, ‘uncertain’ or ‘no’ to each item on the questionnaire. A score of 3 was given for a ‘yes’ response to an item on the scale, 2 for ‘possibly’, 1 for ‘uncertain’ and 0 for ‘no’. A mean value for each item on the scales is then calculated from the participant responses ranging from 0 indicating low stigma to 3 indicating high stigma. This scoring system, and the scales themselves, was developed to aid comparisons rather than establish criteria of stigma or no stigma with a clear numerical cut off point.

### Participant selection

#### Study area and population

The scales were piloted in Wolaita zone, Southern Ethiopia, which has a population of around 1.53 million, 88.3% living in rural areas, the majority working as subsistence farmers [16]. Wolaita zone was chosen because of the high prevalence of podoconiosis (5.5%) [17], and the community’s acceptance and responsiveness to previous podoconiosis research. The Mossy Foot Treatment and Prevention Association (MFTPA, a non-government organisation specialising in podoconiosis prevention and care) also provides a contact base for over 35,000 podoconiosis patients [18].

#### Sample size

Assumptions used in the sample size estimate were drawn from Brieger’s study measuring onchocerciasis (another NTD) stigma in Western Nigeria (Briefer et al., 1998), and the desired size of reduction in stigma level towards podoconiosis patients were an intervention to be introduced.

We used a mean stigma score of 17 and standard deviation of 10 (Brieger’s 13 item, 39 point scale), and the following equation:

$$\mathrm{Use}\;n=\frac{{\left(u+v\right)}^{2*}\left(SD_{12}+SD_{22}\right)}{{\left(\mathrm{mea}n_{1}-\mathrm{mea}n_{2}\right)}^{2}}$$

(where u = 1.28 for power of 90%; v = 1.96 for 2-sided significance level of 5%; SD_1_ = 10, SD_2_ = 10) (Kirkwood 1988).

A series of sample sizes were calculated based on a range of stigma reductions anticipated. A sample size of 150 podoconiosis patients was calculated to give 90% power to detect a stigma reduction of 20–25%. Less dramatic reduction in stigma level was anticipated in the non-affected community, and 500 community controls were calculated to give 90% power to detect a reduction of 10–15%. A sample of 500 unaffected community members and 150 podoconiosis patients is also adequate to enable factor analysis and thereby validate the scales, based on item to case ratio recommendations for factory analyses. Costello and colleagues recommend an optimal sample of 20 individuals to one item [19].

#### Sampling technique

A two stage sampling was used to identify 150 podoconiosis patients in Wolaita Zone. First, five of fourteen active clinic sites of the MFTPA were randomly selected. Systematic sampling was then employed using the patient list at each clinic site to select 30 patients. Any participants who might have been experiencing stigma for other health afflictions such as leprosy or other visible skin diseases were excluded.

Unaffected community members were identified through multistage stratified random sampling. Firstly four *weredas* (districts) were randomly identified and within each *wereda*, two *kebeles* (smallest administrative unit) or Peasant Associations (PAs) were randomly selected. Within each of the *weredas*, 125 adult (≥ 18 years) household heads were systematically selected from the list obtained from the two *kebele* administrative offices to make a total sample size of 500 community members. If any of those individuals identified themselves as having podoconiosis, they were excluded from the sample.

### Data collection

Due to the low literacy rate of participants the questionnaire was interview-based. Six data collectors were fully briefed on the purpose of study, the eligibility criteria for participants and given training on how to request consent and administer the questionnaires.

Data were collected in May 2011. Podoconiosis patients were interviewed in a private area of the clinic they were approached in. Non-affected community members were interviewed in a private area of their home.

All participants were asked to give their responses according to a 12 month time frame (‘since the start of the last rainy season…’) in order to measure recent accounts and perceptions of stigma.When administering the questionnaire, the participant’s age, gender and education level were recorded, and for podoconiosis patients their disease stage was also noted for further analysis.

### Ethical considerations and regulatory approval

Special attention was given to dealing with the sensitive topic of stigma when obtaining informed consent from participants. Following guidance from early studies of the ethics of approaching this community [20], podoconiosis patients were first asked to participate by MFTPA staff with whom they were familiar, before official consent was obtained by data collectors.

Information sheets and consent forms were inapplicable in this particular setting due to the low-literacy level of participants. Instead, the purpose of the study was explained orally, in the style of a conversation rather than reading out an information sheet, and potential participants gave their consent verbally in front of a witness as confirmation. Ethical approval for this study was given by the IRB of Wolaita Sodo University in Ethiopia.

### Data analysis

Before analysis, we decided that since felt stigma and enacted stigma represent two distinct dimensions, they should be assessed through two independent scales. Four scales were therefore analysed in total: a podoconiosis patient felt stigma scale, a community felt stigma scale, a podoconiosis patient enacted stigma scale and a community enacted stigma scale.

The scales were evaluated through reliability assessment, content and construct validity analysis of the items, factor analysis and internal consistency analysis.

#### Reliability assessment

Reliability was assessed through consistency analysis using Cronbach’s alpha. An alpha of 0.7 to 0.9 was considered good consistency [21]. The impact of deleting an item on overall consistency was also assessed, with the aim of discarding an item whenever its deletion led to a noticeable improvement in the overall consistency of the rest of the items.

#### Content and construct validity

Content validity was examined using Spearman’s correlation coefficients. Construct validity was analysed using exploratory factor analysis (FA) with principal components analysis (PCA). Separate factor analyses were performed for felt and enacted stigma for both podoconiosis patients and the community. The first round of un-rotated factor analyses yielded Scree Plots to determine the number of factors underlying the respective stigma dimensions. To find the best fit to the data, orthogonal and non-orthogonal analyses were also conducted. Factor loadings of more than 0.4 were considered satisfactory for the patient and community questionnaires [19]. Findings of the PCA were further validated by split-half reliability analysis, in which the two random halves of the data were expected to provide similar factor solutions. Following recommendations [22,23], factors generated by the PCA were extracted as valid if at least two of the following criteria were met: (1) eigenvalues were equal to or greater than the randomly generated factors from Horn’s parallel analysis; (2) the scree (Cattel’s) test was passed and; (3) eigenvalues of equal or more than unity [24,25].

For analysis on any given variable, cases were excluded if data on that variable were missing. Total scores were only calculated if a case had responded to more than 50% of the items. SPSS 15.0 was used for reliability and validity analysis, while Monte-Carlo PA software was used for parallel analysis. An alpha of 0.05 or less was considered significant.

## Results

### Participant demographics

In the podoconiosis patient sample, all 150 questionnaires were used in analysis. Of the 500 community sample, 17 questionnaires were either incomplete or inadequately completed, and were excluded from analysis, leaving a total sample of 483 (Table 2).

**Table 2:** Participant demographics

**Variables**	**Category**	**Podoconiosis patients**	**Unaffected community members**
		**Number (%)**	**Number (%)**
Sex	Female	81 (54)	204 (42.2)
	Male	69 (46)	279 (57.8)
Age	<25	23 (15.3)	57 (11.8)
	25–34	35 (47)	146 (30.2)
	35–49	47 (31.3)	209 (43.3)
	> = 50	45(30)	71 (14.7)
Education	Illiterate	98 (65.3)	207 (42.9)
	Literate	52 (34.7)	276 (57.1)

### Podoconiosis patients felt stigma scale

In the factor analysis, one item from the preliminary patient questionnaire was rejected because it had loadings below our criterion value of 0.4.

There existed a good consistency between items: and Cronbach's alpha was 0.955. No item significantly improved the level of consistency when deleted. There was modest correlation between items, but no pair of correlations had a value more than 0.9.

PCA revealed a single factor model explaining 61.5% of the data. The first factor, with an eigen value of 9.2, explained about 61.5% of the variance, while the second, with an eigen value of 1.03, explained about 6.9% (Table 3). The first factor met the criterion value generated by parallel analysis, and passed the scree test. The final felt stigma scale for patients comprised 15 items:

Interpersonal interactions

1. Have you avoided taking part in labour or other activities which require group involvement with unaffected people?

2. Have you tried to use household utensils separately from other family members?

3. Have you felt your family are proud of you?

4. Have you continued seeing and spending time with your *(unaffected)* friends?

5. Have you avoided asking neighbours for help, or to borrow items because of your condition?

6. Have you tried to kill yourself because of the demeaning treatment you receive as a result of your condition?

7. Have you tried to change your place of residence because of the way you are treated as a result of your condition?

Major life areas

1. Have you avoided any invitation to be employed for wage labour/job fearing stigma in the work place?

2. Have you feared unaffected individuals may feel uncomfortable working with you because of your condition?

3. Have you avoided marriage to an unaffected person fearing mistreatment after marriage?

4. Have you feared that marriage with an unaffected person may end in divorce?

5. Do you feel your condition has affected your other family member’s chances of marrying?

Community, social and civic life

1. Do you think that your condition deprives you from playing a leadership role in the community?

2. Have you avoided visiting public places like church, school or market?

3. Have you felt able to move around the community freely *(without being stared at, pointed at or people noticing you?)*

**Table 3:** **Patient data: factor extraction decision**^**¥**^

**Components**	**Eigen value**	**Total variance (%)**	**Extraction criteria**				**Decision to extract**
			**PA random eigen value (SD)**		**Kaiser**	**Scree test**	
Felt stigma							
1	9.23	61.5	1.59 (0.07)	Yes	Yes	Yes	Yes
2	1.03	6.9	1.45 (0.05)	No	Yes	No	No
3	0.81	5.0	1.34 (0.04)	No	No	No	No
Enacted stigma							
1	10.7	63.0	1.63 (0.07)	Yes	Yes	Yes	No
2	1.04	6.1	1.49 (0.05)	No	Yes	No	No
3	0.96	5.6	1.39 (0.04)	No	No	No	No

^¥^ The non rotated factor analysis provided the best fit to the data; Kaiser-Meyer-Olkin sampling adequacy equals 0.929 and 0.944 for perceived and enacted stigmas respectively; All anti-image matrices measures of sampling adequacy (MSA) were near or greater than 0.9 for both scales; Bartlett’s test of sphericity, p < 0.001 for both scales; ‘Yes’, indicates criteria is fulfilled, ‘No’ indicates otherwise. Kaiser recommends extracting factors with eigen value of ≥1; Scree (Cattel’s) test recommends extracting factors above the elbow of the scree plot. PA: parallel analysis.

### Podoconiosis patients enacted stigma scale

Cronbach's alpha was 0.94, indicating good consistency between items. No item significantly improved the level of consistency when deleted. There was modest correlation between items, but no pair of correlations had a value more than 0.9.

PCA produced a single factor model explaining 63.0% of the variance. The first factor had an eigen value of 10.7 and explained about 63.0% of the variance, while the second one had an eigen value of 1.04 and explained about 6.1% of the variance (Table 3). The first factor was above the criterion value generated by parallel analysis, and passed the scree test. The final enacted scale for patients is made up of 17 items:

Interpersonal interactions

1. Has anyone in your neighbourhood deterred you from taking part in group activities?

2. Have your family members avoided sharing household utensils with you?

3. Have friends been visiting you less or spending less time with you because of your condition?

4. Have you received insults from others regarding your foot?

Major life areas

1. Is there an example where you or another person you know has been denied a job opportunity because of the condition?

2. Have you or another person you know been forced to leave a job because of the condition?

3. Have you or another person you know been mistreated at your work place due to the condition?

4. Have you or another person you know been forced to dissolve marriage plans because of the condition?

5. Have you or another person you know experienced divorce because of the condition?

6. Has your condition made it difficult for an unaffected member of your family to marry?

Community, social and civic life

1. Is there an example where you or another person you know has been denied the chance of a leadership role due to the condition?

2. Is there an example where you or another person you know have been denied the chance to make decisions in community matters?

3. Have people ignored you, talked over you or told you to be quiet because of your condition?

4. Have you or another person you know been welcomed *(by unaffected people)* while attending church, school or other community meeting places?

5. Is there an example where you or another person you know have been treated in isolation at a social event?

6. Is there an example where you or another person you know have not been invited to appear at public places?

7. Is there an example where you or another person you know has been stared or pointed at when attending a social event?

### Unaffected community felt stigma scale

In the factor analysis, one item from the preliminary community questionnaire was rejected because it had loadings below our criterion value of 0.4.

Cronbach’s alpha was 0.85, indicating good consistency between items. No item significantly improved the level of consistency when deleted. There was modest amount of correlation between items, but no pair of correlations had a value more than 0.9.

PCA provided a four factor model explaining 49.4% of the data. The first factor had an eigen value of 5.8 and explained about 24% of the variance, while the second had an eigen value of 2.8, and explained about 11.7%. These were above the criterion value generated by parallel analysis, and fulfil the scree test criteria (Table 4). These analyses were also valid when re-run using random halves of the dataset. The final felt stigma scale for the community comprised 24 items:

Interpersonal interactions

1. Have you seen or heard of someone with this condition who fears bringing items to the market thinking they may not be sold?

2. Have you seen or heard of someone with this condition hesitating to visit a health centre fearing that health professionals may pity them?

3. Have you seen or heard about the children of someone with this condition dropping out school?

4. Have you seen or heard of someone with this condition avoiding taking part in group activities such as labour with unaffected people?

5. Have you seen or heard of someone with this condition trying to use household utensils separately from other family members?

6. Have you seen or heard of someone with this condition avoiding preparing food for other family members?

7. Does someone with this condition feel their family is proud of them?

8. Do you think someone with this condition is happy seeing or spending time with unaffected friends?

9. Do you think someone with this condition feels comfortable asking neighbours for help, or to borrow items?

10. Have you seen or heard of someone with this condition who has committed or tried to commit suicide?

11. Have you seen or heard of someone with this condition who has changed or tried to change the place where they live?

Major life areas

1. Have you seen or heard of someone with this condition who feels uncomfortable working with unaffected individuals?

2. Do you think someone with this condition feels they are as capable or productive as others?

3. Have you seen or heard of someone with this condition who has avoided marrying an unaffected person fearing mistreatment after marriage?

4. Does someone with this condition feel confident asking an unaffected person for marriage?

5. Do you think someone with this condition fears that marriage to an unaffected person might end in divorce?

6. Do you think someone with this condition feels their condition has affected other family member’s chances of marrying?

Community, social and civic life

1. Do you think someone with this condition feels comfortable participating in community affairs?

2. Is someone with this condition afraid to accept a leadership offer or play a leadership role in the community?

3. Do you think someone with this condition is afraid to take part in decision making fearing their ideas might be discredited?

4. Does someone with this condition feel confident appearing at public places?

5. Have you seen or heard about someone with this condition who has avoided visiting public places like church, school or market?

6. Do you think that unaffected people and people with this condition should be served separately in public places?

7. Do you think someone with this condition fears others will stare or point at them in public places?

**Table 4:** **Community data: factor extraction decision**^**¥**^

**Components**	**Eigen value**	**Total variance (%)**	**Extraction criteria**				**Decision to extract**
			**PA random eigen value (SD)**		**Kaiser**	**Scree test**	
Felt stigma							
1	5.79	24.10	1.42 (.04)	Yes	Yes	Yes	Yes
2	2.81	11.7	1.35 (.03)	Yes	Yes	Yes	Yes
3	1.75	7.3	1.30 (.02)	Yes	Yes	Yes	Yes
4	1.50	6.3	1.26 (.02)	Yes	Yes	No	Yes
Enacted stigma							
1	7.84	34.1	1.4075 (.04)	Yes	Yes	Yes	Yes
2	1.97	8.6	1.3411 (.04)	Yes	Yes	Yes	Yes
3	1.27	5.5	1.2915 (.02)	No	Yes	No	No
4	1.20	5.2	1.2473 (.02)	No	Yes	No	No

^¥^ The non rotated factor analysis provided the best fit to the data; Kaiser-Meyer-Olkin sampling adequacy equals 0.85 and 0.90 for perceived and enacted stigmas respectively; All anti-image matrices measures of sampling adequacy (MSA) were near or greater than 0.90 for both scales; Bartlett’s test of sphericity, p < 0.000 for both scales; ‘Yes’, indicates criteria is fulfilled, ‘No’ indicates otherwise. Kaiser recommends extracting factors with eigen value of ≥1; Scree (Cattel’s) test recommends extracting factors above the elbow of the scree plot. PA: parallel analysis.

### Unaffected community enacted stigma scale

Two items (one related to being interested in eating food prepared by someone with podoconiosis and the other concerning podoconiosis patients being welcomed at community meeting places) were rejected from the preliminary questionnaire because they had loadings below our criterion value of 0.4.

Cronbach’s alpha was 0.91, indicating good consistency between items. No item significantly improved the level of consistency when deleted. There was modest amount of correlation between items, but no pair of correlations had a value more than 0.9.

PCA produced a two factor model explaining 42.6% of the variance. The first factor, with an eigen value of 7.8, explained about 34% of the variance, while the second, with an eigen value of 1.96, explained about 8.6%. These were above the criterion value generated by parallel analysis, and fulfil the scree test criteria (Table 4). These analyses were also valid when re-run using random halves of the dataset. The final enacted stigma scale for the community is made up of 23 items:

Interpersonal interactions

1. Has someone with this condition selling items at market been avoided?

2. Have you seen or heard of anyone with this condition being pitied by a health professional?

3. Have you seen or heard of any children of someone with this condition being mistreated by friends or teachers at school?

4. Has someone with this condition been stopped from taking part in group activities in your neighbourhood?

5. Have people avoided sharing or borrowing household utensils with someone with this condition?

6. Have people avoided sitting near or sharing a seat with someone with this condition?

7. Have you seen or heard about anyone with this condition being mistreated by their family compared to unaffected members?

8. Have you seen or heard about someone with this condition being isolated by their friends, family members or neighbours?

9. Have you seen or heard insults being called out to someone with this condition?

Major life areas

1. Do you know of anyone who has not been offered a job because of this condition?

2. Have you seen or heard of anyone who was forced to leave their job because of this condition?

3. Have you seen or heard of anyone who has been mistreated in the work place because of this condition?

4. Have you seen or heard of anyone who has avoided marriage with someone because they had this condition?

5. Have you seen or heard of anyone who has been divorced because of this condition?

6. Have you seen or heard of anyone who has been mistreated by their by spouse due to this condition?

7. Have you seen or heard of someone where their condition made it difficult for their unaffected family members to marry?

Community, social and civic life

1. Has there been an example where someone has been denied the chance of a leadership role because of this condition?

2. Has there been an example where someone with this condition has been denied the chance to make decisions in community matters?

3. Has there been an example where someone with this condition has not been listened too or ignored when talking?

4. Has there been an example where someone with this condition has not been invited to appear at public places?

5. Has someone with this condition been isolated at a social event you have attended?

6. Has someone with this condition been stared or pointed at while attending a social event?

7. Have you experienced someone with this condition being excluded from using public facilities, such as public transport?

## Discussion and conclusions

### Strengths

We report the development and testing of the first standardised measures of podoconiosis stigma. All scales had Cronbach’s alpha over 0.7, indicating good internal consistency. The construct and discriminant validity of the scales were satisfactory with modest correlation between items. There was significant correlation between the felt stigma scale and enacted stigma scale among patients (Spearman's r = 0.892; p = 0.000; N = 150) and within the community (Spearman's r = 0.794; p = 0.000).

Four scales have been produced which may be used individually as single instruments to measure specific aspects of podoconiosis stigma for certain social groups, or may be combined to gain a more extensive overview of the level of stigma towards podoconiosis in a given community. The tools assessed both felt and enacted stigma across three domains which covered both public and private settings. Items are framed in order to record actual rather than hypothetical acts of stigma over a specific time frame which will aid comparison of the level of stigma pre- and post-intervention.

The greatest strength of this study is that it will enable an evidence based approach to mitigating stigma, identifying specific areas to target based on the precise needs of the community. As Hotez [7] points out, there is no ‘one size fits all’ method for stigma reduction.

### Limitations

One potential limitation of the study is the effect of social desirability bias which may have compromised participants’ report of prejudicial attitudes and discriminatory behaviours, particularly since the questionnaires were interview-based rather than self-administered. Given the low literacy of the population, we had no alternative but to use interviews to gather data, and we acknowledge that this may have reduced the reliability of the findings.

The recruitment of podoconiosis patients through MFTPA clinics may have introduced selection bias, possibly resulting in exclusion of the most stigmatised patients who may not have reached these clinics.

The scales require further testing in other contexts in order to validate them. Correlations between these scales and others measuring variables related to stigma (such as self-esteem and depression) or quality of life might also be assessed.

### Future uses of the scales

The scales developed as a result of this study will increase understanding of the dynamics of podoconiosis stigma and in turn, enable the design of more effective reduction strategies [2,26].

We envisage the scales’ primary function to be in situational analysis where they can be used to assess the state of the population before intervention. Through use of the scales, areas of stigma which are most profound and those social groups which are particularly affected by stigma can be identified. The data collected can then be used in the preparation and planning of stigma reduction strategies. The same scales may also be used to monitor and evaluate stigma interventions and to compare the efficacy of different strategies [26].

The four scales will also benefit future research on podoconiosis stigma and in turn, prevention and treatment of the disease as a whole. Furthermore, the scales can be used to compare stigma intensity between different groups or communities and discover factors that increase the risk of stigmatisation from podoconiosis.

The ability to quantify podoconiosis stigma will help justify interventions to reduce it and mobilise further action [26]. Data produced by the scales can strengthen the case of people involved in advocacy on behalf of those stigmatised as a result of podoconiosis, such as the MFTPA. The information collected can be used to generate momentum towards tackling podoconiosis and highlight the severity of the stigma burden, helping to bring podoconiosis to government attention as a public health issue.

In conclusion, we have developed four reliable and valid scales to measure stigma towards podoconiosis and guide interventions targeted towards reducing the burden. Continued research that documents the impact of NTD stigma on health-seeking behaviour and disease control is required. The development of scales to assess stigma are particularly valuable when applied to NTDs since the stigma associated with diseases such as podoconiosis compound the effects of poverty. Collecting data that can guide policies and programs which seek to mitigate the constraints of stigma allow individuals to relieve themselves of the conditions that cause NTDs to persist.

## Competing interests

The authors declared that they have no competing interest.

## Authors’ contributions

HF, AT and GD designed the study. AT and HF coordinated and oversaw the data collection. AAR and KD conducted the statistical analysis. All authors contributed to the discussion of results. HF produced the first draft of the manuscript and all authors reviewed and approved the final manuscript.

## Pre-publication history

The pre-publication history for this paper can be accessed here:

http://www.biomedcentral.com/1471-2458/13/298/prepub
